# HLA Class I and Class II Conserved Extended Haplotypes and Their Fragments or Blocks in Mexicans: Implications for the Study of Genetic Diversity in Admixed Populations

**DOI:** 10.1371/journal.pone.0074442

**Published:** 2013-09-23

**Authors:** Joaquín Zúñiga, Neng Yu, Rodrigo Barquera, Sharon Alosco, Marina Ohashi, Tatiana Lebedeva, Víctor Acuña-Alonzo, María Yunis, Julio Granados-Montiel, Alfredo Cruz-Lagunas, Gilberto Vargas-Alarcón, Tatiana S. Rodríguez-Reyna, Marcelo Fernandez-Viña, Julio Granados, Edmond J. Yunis

**Affiliations:** 1 Department of Immunology, Instituto Nacional de Enfermedades Respiratorias Ismael Cosío Villegas, Mexico City, Mexico; 2 HLA Laboratory, The American Red Cross Northeast Division, Dedham, Massachusetts, United States of America; 3 Molecular Genetics Laboratory, National School of Anthropology and History, Mexico City, Mexico; 4 Department of Cancer Immunology and AIDS, Dana Farber Cancer Institute, Harvard Medical School, Boston, Massachusetts, United States of America; 5 Tissue Engineering, Cell Therapy and Regenerative Medicine Research Unit, Instituto Nacional de Rehabilitación, Mexico City, Mexico; 6 Laboratory of Genomics, Instituto Nacional de Cardiología Ignacio Chavez, Mexico City, Mexico; 7 Department of Immunology and Rheumatology, Instituto Nacional de Ciencias Médicas y Nutrición Salvador Zubirán, Mexico City, Mexico; 8 Department of Pathology, Stanford University, Stanford, California, United States of America; 9 Department of Transplantation, Instituto Nacional de Ciencias Médicas y Nutrición Salvador Zubirán, Mexico City, Mexico; University of Hawaii Manoa, United States of America

## Abstract

Major histocompatibility complex (MHC) genes are highly polymorphic and informative in disease association, transplantation, and population genetics studies with particular importance in the understanding of human population diversity and evolution. The aim of this study was to describe the HLA diversity in Mexican admixed individuals. We studied the polymorphism of MHC class I (*HLA-A*, *-B*, *-C*), and class II (*HLA-DRB1*, *-DQB1*) genes using high-resolution sequence based typing (SBT) method and we structured the blocks and conserved extended haplotypes (CEHs) in 234 non-related admixed Mexican individuals (468 haplotypes) by a maximum likelihood method. We found that HLA blocks and CEHs are primarily from Amerindian and Caucasian origin, with smaller participation of African and recent Asian ancestry, demonstrating a great diversity of HLA blocks and CEHs in Mexicans from the central area of Mexico. We also analyzed the degree of admixture in this group using short tandem repeats (STRs) and *HLA-B* that correlated with the frequency of most probable ancestral *HLA-C*/−*B* and *-DRB1*/−*DQB1* blocks and CEHs. Our results contribute to the analysis of the diversity and ancestral contribution of HLA class I and HLA class II alleles and haplotypes of Mexican admixed individuals from Mexico City. This work will help as a reference to improve future studies in Mexicans regarding allotransplantation, immune responses and disease associations.

## Introduction

The human major histocompatibility complex (MHC) is located within chromosomal region 6p21.3 and spans at least 3.4 Mb of DNA containing as many as 420 genes, including the HLA system, other immune related genes and pseudogenes [1]. The extensive polymorphism of the HLA genes within populations could have resulted from selective pressures including functional adaptation particularly to bacteria, viruses and parasites [2]–[5]. Also, the hypothesis of heterozygote advantage proposed that individuals with heterozygosity at HLA loci would be more efficient to respond against pathogens in pathogen-enriched environments [6]. Nevertheless, studies of genetics of infectious diseases are difficult to replicate due to the complex nature of the environmental factors and the degree of genetic diversity among human populations. In this regard, MHC genes are important because they are involved in immune responses, and are essential markers to study genetic diversity, disease susceptibility and allotransplantation [7].

Different studies using DNA polymorphic markers such as short tandem repeats (STRs), low and intermediate resolution HLA typing, ABO, MN and Rr-Hr blood groups, serum haptoglobin, albumin, and Factor Bf types have described the complexity of the genetic admixture of Mexican populations. These studies have revealed a non-homogeneus combination of Amerindian, Caucasian, and African genes in Mexican admixed individuals [8]–[10]. In this context, an important role of ethnicity in the susceptibility to different inflammatory and infectious diseases has been attributable to the incorporation of MHC alleles by admixture with Caucasian, Asian and African populations [11].

An important aspect of the MHC genetics is the inheritance of non-random associated alleles known as linkage disequilibrium (LD) [12]. Extensive studies on the existence of small blocks and other relatively fixed genetic fragments within the human MHC have been conducted [7], [13]. Specific DNA blocks with specific alleles of two or more MHC loci are often haplospecific for particular conserved extended haplotypes (CEHs). The frequency of CEHs and specific block combinations varies between major ethnic groups and/or in different geographic locations; these variations in the frequency of CEHs and blocks can be used as measurements of genetic diversity of the MHC [13]; however, little is known about the MHC blocks distribution and conserved haplotypes combination in Latin-American admixed human groups. Thus, the aim of the present study is to describe the distribution of HLA class I and class II blocks and the HLA CEHs using high resolution typing in a group of Mexican admixed individuals from Mexico City.

## Results

### *HLA-A*, *-B*, *-C*, *-DRB1*, and *-DQB1* Allelic Frequencies in Mexican Admixed Individuals

The distribution of *HLA-A*, *-B*, *-C*, *-DRB1* and *-DQB1* alleles are listed in **Table 1**. We detected 34 *HLA-A*, 64 *HLA-B*, 28 *HLA-C*, 39 *HLA-DRB1*, and 15 *HLA-DQB1* alleles. The most frequent alleles were: **1)** ***HLA-A*** **:** A*02∶01, A*24∶02, A*02∶06. A*68∶01, and A*31∶01, **2)** ***HLA-B*** **:** B*39∶05, B*39∶06, B*51∶01, B*35∶01, and B*40∶02; **3)** ***HLA-C*** **:** C*07∶02, C*04∶01, C*01∶02, C*03∶04, C*06∶02, and C*07∶01; **4)** ***HLA-DRB1*** **:** DRB1*08∶02, DRB1*04∶07, DRB1*14∶06, DRB1*07∶01, DRB1*04∶04, and DRB1*16∶02 and **5)** ***HLA-DQB1*** **:** DQB1*03∶01, DQB1*03∶02, DQB1*04∶02, DQB1*05∶01, and DQB1*02∶02 with frequencies higher than 5%. A significant deviation from Hardy-Weinberg equilibrium (HWE) was detected at the HLA-DRB1 locus (p<0.05).

**Table 1:** Allelic frequencies of *HLA-A, -B, -C, -DRB1*, and *-DQB1* in 234 Mexicans.

HLA-A	F	HLA-B	F	HLA-C	F	HLA-DRB1	F	HLA-DQB1	F
A*02∶01	0.2286	B*39∶05	0.0791	C*07∶02	0.2073	DRB1*08∶02	0.1944	DQB1*03∶01	0.2479
A*24∶02	0.1688	B*39∶06	0.0684	C*04∶01	0.1859	DRB1*04∶07	0.1175	DQB1*03∶02	0.2457
A*02∶06	0.0962	B*51∶01	0.0598	C*01∶02	0.0897	DRB1*14∶06	0.1004	DQB1*04∶02	0.2051
A*68∶01	0.0791	B*35∶01	0.0577	C*03∶04	0.0662	DRB1*07∶01	0.0705	DQB1*05∶01	0.0684
A*31∶01	0.0791	B*40∶02	0.0534	C*06∶02	0.0598	DRB1*04∶04	0.0662	DQB1*02∶02	0.0598
A*01∶01	0.0363	B*48∶01	0.0427	C*07∶01	0.0534	DRB1*16∶02	0.0641	DQB1*06∶02	0.0363
A*68∶03	0.0342	B*07∶02	0.0406	C*08∶01	0.0470	DRB1*15∶01	0.0363	DQB1*02∶01	0.0321
A*03∶01	0.0321	B*35∶17	0.0385	C*08∶02	0.0406	DRB1*03∶01	0.0321	DQB1*03∶03	0.0214
A*68∶02	0.0299	B*35∶12	0.0385	C*03∶05	0.0342	DRB1*13∶01	0.0256	DQB1*06∶04	0.0214
A*29∶02	0.0256	B*14∶02	0.0321	C*03∶03	0.0299	DRB1*01∶02	0.0235	DQB1*05∶03	0.0171
A*11∶01	0.0214	B*15∶15	0.0321	C*16∶01	0.0256	DRB1*14∶02	0.0235	DQB1*06∶03	0.0150
A*26∶01	0.0192	B*44∶03	0.0278	C*12∶03	0.0256	DRB1*04∶03	0.0214	DQB1*06∶01	0.0107
A*23∶01	0.0171	B*52∶01	0.0214	C*15∶09	0.0235	DRB1*13∶02	0.0214	DQB1*03∶19	0.0107
A*02∶05	0.0171	B*15∶01	0.0214	C*05∶01	0.0214	DRB1*04∶02	0.0214	DQB1*05∶02	0.0064
A*30∶02	0.0150	B*39∶02	0.0214	C*15∶02	0.0192	DRB1*04∶11	0.0192	DQB1*05:new†	0.0021
A*30∶01	0.0128	B*35∶43	0.0192	C*02∶02	0.0128	DRB1*01∶01	0.0192		
A*33∶01	0.0128	B*49∶01	0.0192	C*14∶02	0.0085	DRB1*11∶04	0.0171		
A*66∶01	0.0128	B*18∶01	0.0171	C*08∶03	0.0085	DRB1*14∶01	0.0171		
A*32∶01	0.0085	B*15∶30	0.0171	C*16∶04	0.0043	DRB1*11∶01	0.0128		
A*24∶25	0.0064	B*57∶01	0.0150	C*02∶10	0.0043	DRB1*10∶01	0.0128		
A*25∶01	0.0064	B*35∶14	0.0150	C*03∶02	0.0043	DRB1*15∶02	0.0107		
A*68∶05	0.0064	B*13∶02	0.0128	C*12∶02	0.0043	DRB1*11∶02	0.0085		
A*03∶02	0.0064	B*53∶01	0.0128	C*04∶07	0.0043	DRB1*12∶02	0.0064		
A*01∶02	0.0043	B*38∶01	0.0128	C*16∶02	0.0021	DRB1*13∶03	0.0064		
A*33∶03	0.0043	B*58∶02	0.0128	C*07∶04	0.0021	DRB1*04∶01	0.0064		
A*02∶211	0.0021	B*39∶01	0.0107	C*15∶05	0.0021	DRB1*01∶03	0.0064		
A*34∶01	0.0021	B*40∶05	0.0107	C*17∶01	0.0021	DRB1*16∶01	0.0043		
A*01∶03	0.0021	B*35∶03	0.0107	C*03:new§	0.0021	DRB1*04∶10	0.0043		
A*34∶02	0.0021	B*41∶01	0.0107			DRB1*08∶04	0.0043		
A*30∶04	0.0021	B*44∶02	0.0107			DRB1*12∶01	0.0043		
A*74∶01	0.0021	B*35∶24	0.0085			DRB1*13∶05	0.0021		
A*26∶17	0.0021	B*37∶01	0.0085			DRB1*04∶05	0.0021		
A*02∶02	0.0021	B*14∶01	0.0085			DRB1*15∶03	0.0021		
A*02∶24	0.0021	B*50∶01	0.0085			DRB1*08∶03	0.0021		
		B*40∶27	0.0085			DRB1*04∶08	0.0021		
		B*55∶01	0.0064			DRB1*03∶02	0.0021		
		B*58∶01	0.0064			DRB1*08∶01	0.0021		
		B*45∶01	0.0064			DRB1*09∶01	0.0021		
		B*27∶05	0.0064			DRB1*13∶04	0.0021		
		B*08∶01	0.0064						
		B*39∶08	0.0064						
		B*35∶08	0.0064						
		B*35∶16	0.0064						
		B*15∶17	0.0064						
		B*51∶02	0.0043						
		B*15∶02	0.0043						
		B*15∶03	0.0043						
		B*39∶10	0.0043						
		B*15∶39	0.0043						
		B*15∶31	0.0043						
		B*35∶02	0.0043						
		B*40∶04	0.0021						
		B*15∶16	0.0021						
		B*40∶20	0.0021						
		B*40∶08	0.0021						
		B*15∶18	0.0021						
		B*15∶10	0.0021						
		B*57∶03	0.0021						
		B*27∶03	0.0021						
		B*07∶14	0.0021						
		B*35∶20	0.0021						
		B*56∶01	0.0021						
		B*35∶40N	0.0021						
		B*35:new*	0.0021						

* Similar to B*35∶01 with a mutation at codon 207 ggc>tgc (Gly>Cys).

§ Similar to C*03∶04 with a mutation at codon 189 gtg>atg (Val>Met).

† Similar to DQB1*05∶02 with a silent mutation at codon 133 cgg>cga.

### Distribution of *HLA-C*/−*B* and *-DRB1*/−*DQB1* Blocks in Mexican Admixed Individuals

*HLA-C/−B* blocks found in this group of Mexican admixed individuals are grouped in **Table 2**. Twenty-six Amerindian (Native American) most probable ancestry (MPA) *HLA-C/−B* blocks (41.3%) were found. The most frequent (frequency ≥3.0%) Amerindian *HLA-C/−B* blocks were: C*07∶02/B*39∶05 (Haplotype Frequency (HF) = 0.0726), C*07∶02/B*39∶06 (HF = 0.0619), C*04∶01/B*35∶17 (HF = 0.0363), C*04∶01/B*35∶12 (HF = 0.0341) and C*08∶01/B*48∶01 (HF = 0.0320).

**Table 2:** Frequencies of *HLA-C-B* blocks in 234 admixed Mexican individuals (468 haplotypes).

	-C-B block		n	H.F.	Δ′	t		-C-B block			n		H.F.		Δ′		t	
**Amerindian**	**C** * **07∶02**	**B** * **39∶05**	34	0.0726	0.8975	6.36	**Asian**	**C** * **04∶01**	**B** * **35∶16**		3		0.0064		1.0000		1.80	
	**C** * **07∶02**	**B** * **39∶06**	29	0.0619	0.8025	5.40		**C** * **14∶02**	**B** * **51∶01**		3		0.0064		0.7339		1.71	
	**C** * **04∶01**	**B** * **35∶17**	17	0.0363	1.0000	4.43		**C** * **08∶01**	**B** * **15∶02**		2		0.0042		1.0000		1.42	
	**C** * **04∶01**	**B** * **35∶12**	16	0.0341	0.8632	4.14		**C** * **07∶04**	**B** * **15∶18**		1		0.0021		1.0000		1.00	
	**C** * **08∶01**	**B** * **48∶01**	15	0.0320	0.7376	4.02		**C** * **03∶03**	**B** * **35∶01**		1		0.0021		0.0141		0.19	
	**C** * **03∶04**	**B** * **40∶02**	11	0.0235	0.4196	3.20		**C** * **01∶02**	**B** * **35∶01**		1		0.0021		−0.5908		−1.21	
	**C** * **03∶05**	**B** * **40∶02**	10	0.0213	0.6045	3.19		**C** * **12∶03**	**B** * **35∶03**		1		0.0021		0.1788		0.90	
	**C** * **01∶02**	**B** * **15∶30**	8	0.0170	1.0000	2.92		**C** * **07∶02**	**B** * **40∶02**		1		0.0021		−0.8007		−2.17	
	**C** * **01∶02**	**B** * **15∶01**	7	0.0149	0.6701	2.61		**C** * **04∶01**	**B** * **40∶05**		1		0.0021		0.0154		0.06	
	**C** * **03∶04**	**B** * **39∶02**	5	0.0106	0.4642	2.13		**C** * **01∶02**	**B** * **55∶01**		1		0.0021		0.2670		0.79	
	**B** * **35∶01**	**C** * **03∶05**	5	0.0106	0.2700	1.98		**C** * **03∶02**	**B** * **58∶01**		1		0.0021		0.4967		1.00	
	**C** * **04∶01**	**B** * **35∶03**	4	0.0085	0.7538	1.93	**Total**			**16**		**0.0338**						
	**C** * **04∶01**	**B** * **35∶24**	4	0.0085	1.0000	2.10												
	**C** * **07∶02**	**B** * **39∶01**	4	0.0085	0.7471	1.93	**Unknown**	**C** * **01∶02**	**B** * **15∶15**		13		0.0277		0.8534		3.71	
	**C** * **07∶02**	**B** * **39∶02**	4	0.0085	0.2414	1.12		**C** * **01∶02**	**B** * **35∶43**		9		0.0192		1.0000		3.10	
	**C** * **03∶04**	**B** * **40∶05**	3	0.0064	0.5714	1.66		**C** * **15∶09**	**B** * **51∶01**		9		0.0192		0.8065		3.06	
	**C** * **07∶01**	**B** * **15∶17**	3	0.0064	1.0000	1.75		**C** * **04∶01**	**B** * **35∶14**		6		0.0128		1.0000		2.58	
	**C** * **07∶02**	**B** * **39∶08**	3	0.0064	1.0000	1.83		**C** * **03∶03**	**B** * **52∶01**		6		0.0128		0.5876		2.47	
	**C** * **08∶03**	**B** * **48∶01**	3	0.0064	0.7387	1.72		**C** * **04∶01**	**B** * **35∶08**		3		0.0064		1.0000		1.80	
	**C** * **02∶02**	**B** * **27∶05**	2	0.0042	0.6623	1.41		**C** * **03∶04**	**B** * **40∶27**		3		0.0064		0.7321		1.71	
	**C** * **08∶01**	**B** * **51∶02**	2	0.0042	1.0000	1.42		**C** * **06∶02**	**B** * **07∶02**		2		0.0042		0.0540		0.68	
	**C** * **07∶02**	**B** * **35∶01**	1	0.0021	−0.8228	−2.37		**C** * **08∶01**	**B** * **14∶01**		2		0.0042		−1.0000		−1.28	
	**C** * **03∶04**	**B** * **35∶01**	1	0.0021	−0.4456	−0.73		**C** * **04∶07**	**B** * **15∶31**		2		0.0042		1.0000		1.42	
	**C** * **03∶05**	**B** * **39∶06**	1	0.0021	−0.0938	−0.10		**C** * **03∶03**	**B** * **15∶39**		2		0.0042		1.0000		1.42	
	**C** * **03∶04**	**B** * **40∶08**	1	0.0021	1.0000	1.00		**C** * **06∶02**	**B** * **35∶02**		2		0.0042		1.0000		1.42	
	**C** * **03∶04**	**B** * **51∶01**	1	0.0021	−0.4654	−0.79		**C** * **07∶02**	**B** * **51∶01**		2		0.0042		−0.6583		−1.85	
**Total**			**194**	**0.4133**				**C** * **15∶05**	**B** * **07∶02**		1		0.0021		1.0000		1.00	
								**C** * **07∶01**	**B** * **07∶14**		1		0.0021		1.0000		1.00	
**Caucasian**	**C** * **07∶02**	**B** * **07∶02**	15	0.0320	0.7893	3.91		**C** * **03∶03**	**B** * **13∶02**		1		0.0021		0.1407		0.85	
	**C** * **16∶01**	**B** * **44∶03**	8	0.0170	0.6571	2.89		**C** * **05∶01**	**B** * **14∶02**		1		0.0021		0.0699		0.70	
	**C** * **12∶03**	**B** * **38∶01**	6	0.0128	1.0000	2.50		**C** * **08∶03**	**B** * **14∶02**		1		0.0021		0.2249		0.90	
	**C** * **05∶01**	**B** * **18∶01**	5	0.0106	0.6167	2.26		**C** * **02∶10**	**B** * **14∶02**		1		0.0021		0.4833		0.97	
	**C** * **05∶01**	**B** * **44∶02**	4	0.0085	0.7956	2.03		**C** * **03∶03**	**B** * **15∶01**		1		0.0021		0.0720		0.72	
	**C** * **06∶02**	**B** * **50∶01**	4	0.0085	1.0000	2.04		**C** * **08∶02**	**B** * **15∶03**		1		0.0021		0.4787		0.96	
	**C** * **07∶01**	**B** * **08∶01**	3	0.0064	1.0000	1.75		**C** * **03∶02**	**B** * **15∶10**		1		0.0021		1.0000		1.00	
	**C** * **06∶02**	**B** * **37∶01**	3	0.0064	0.7339	1.71		**C** * **04∶01**	**B** * **15∶15**		1		0.0021		−0.6444		−1.31	
	**C** * **04∶01**	**B** * **44∶03**	3	0.0064	0.0533	0.34		**C** * **07∶02**	**B** * **15∶15**		1		0.0021		−0.6811		−1.43	
	**C** * **03∶04**	**B** * **15∶01**	2	0.0042	0.1427	1.00		**C** * **08∶02**	**B** * **18∶01**		1		0.0021		0.0876		0.70	
	**C** * **12∶03**	**B** * **18∶01**	2	0.0042	0.2301	1.32		**C** * **01∶02**	**B** * **27∶05**		1		0.0021		0.2670		0.79	
	**C** * **04∶01**	**B** * **39∶06**	2	0.0042	−0.6667	−1.94		**C** * **16∶01**	**B** * **35∶01**		1		0.0021		0.0267		0.31	
	**C** * **06∶02**	**B** * **57∶01**	2	0.0042	0.2398	1.19		**C** * **15∶09**	**B** * **35∶01**		1		0.0021		0.0347		0.37	
	**C** * **02∶02**	**B** * **14∶02**	1	0.0021	0.1388	0.84		**C** * **07∶01**	**B** * **35∶01**		1		0.0021		−0.3126		−0.43	
	**C** * **17∶01**	**B** * **41∶01**	1	0.0021	1.0000	1.00		**C** * **03∶03**	**B** * **35∶12**		1		0.0021		0.0340		0.47	
	**C** * **07∶01**	**B** * **51∶01**	1	0.0021	−0.3371	−0.48		**C** * **07∶02**	**B** * **35∶12**		1		0.0021		−0.7342		−1.71	
	**C** * **04∶01**	**B** * **56∶01**	1	0.0021	1.0000	1.04		**C** * **04∶01**	**B** * **35∶20**		1		0.0021		1.0000		1.04	
	**C** * **07∶02**	**B** * **57∶01**	1	0.0021	−0.3166	−0.41		**C** * **15∶09**	**B** * **35∶40N**		1		0.0021		1.0000		1.00	
**Total**			**64**	**0.1359**				**C** * **03∶04**	**B** * **35:New** *		1		0.0021		1.0000		1.00	
								**C** * **03∶03**	**B** * **37∶01**		1		0.0021		0.2267		0.91	
**Caucasian shared with other populations**	**C** * **04∶01**	**B** * **35∶01**	15	0.0320	0.4530	3.23		**C** * **03∶04**	**B** * **39∶01**		1		0.0021		0.1427		0.71	
	**C** * **08∶02**	**B** * **14∶02**	11	0.0235	0.7219	3.40		**C** * **03:New** §	**B** * **39∶02**		1		0.0021		1.0000		1.00	
	**C** * **15∶02**	**B** * **51∶01**	9	0.0192	1.0000	3.09		**C** * **04∶01**	**B** * **39∶05**		1		0.0021		−0.8559		−2.83	
	**C** * **07∶01**	**B** * **49∶01**	6	0.0128	0.6477	2.45		**C** * **01∶02**	**B** * **39∶05**		1		0.0021		−0.7014		−1.75	
	**C** * **06∶02**	**B** * **13∶02**	5	0.0106	0.8226	2.26		**C** * **02∶02**	**B** * **39∶05**		1		0.0021		0.0945		0.55	
	**C** * **08∶02**	**B** * **14∶01**	4	0.0085	1.0000	2.04		**C** * **03∶04**	**B** * **39∶06**		1		0.0021		−0.5323		−0.99	
	**C** * **07∶01**	**B** * **41∶01**	4	0.0085	0.7886	2.01		**C** * **07∶01**	**B** * **39∶06**		1		0.0021		−0.4200		−0.67	
	**C** * **12∶02**	**B** * **52∶01**	2	0.0042	1.0000	1.42		**C** * **04∶01**	**B** * **40∶02**		1		0.0021		−0.7778		−2.06	
	**C** * **02∶02**	**B** * **40∶02**	1	0.0021	0.1212	0.72		**C** * **07∶02**	**B** * **40∶04**		1		0.0021		1.0000		1.05	
	**C** * **03∶03**	**B** * **55∶01**	1	0.0021	0.3126	0.94		**C** * **07∶02**	**B** * **40∶05**		1		0.0021		−0.0433		−0.04	
**Total**			**58**	**0.1235**				**C** * **03∶04**	**B** * **40∶20**		1		0.0021		1.0000		1.00	
								**C** * **16∶04**	**B** * **44∶02**		1		0.0021		0.4946		0.99	
**African**	**C** * **04∶01**	**B** * **53∶01**	6	0.0128	1.0000	2.58		**C** * **08∶01**	**B** * **44∶03**		1		0.0021		0.0310		0.40	
	**C** * **06∶02**	**B** * **58∶02**	6	0.0128	1.0000	2.51		**C** * **16∶02**	**B** * **44∶03**		1		0.0021		1.0000		1.00	
	**C** * **07∶01**	**B** * **57∶01**	3	0.0064	0.3960	1.62		**C** * **16∶01**	**B** * **48∶01**		1		0.0021		0.0420		0.50	
	**C** * **12∶03**	**B** * **39∶10**	2	0.0042	1.0000	1.42		**C** * **08∶02**	**B** * **48∶01**		1		0.0021		0.0100		0.18	
	**C** * **06∶02**	**B** * **45∶01**	2	0.0042	0.6453	1.38		**C** * **01∶02**	**B** * **49∶01**		1		0.0021		0.0226		0.19	
	**C** * **02∶10**	**B** * **15∶03**	1	0.0021	0.4978	1.00		**C** * **06∶02**	**B** * **49∶01**		1		0.0021		0.0540		0.47	
	**C** * **14∶02**	**B** * **15∶16**	1	0.0021	1.0000	1.00		**C** * **07∶02**	**B** * **49∶01**		1		0.0021		−0.4685		−0.72	
	**C** * **02∶02**	**B** * **27∶03**	1	0.0021	1.0000	1.00		**C** * **08∶02**	**B** * **51∶01**		1		0.0021		−0.1278		−0.14	
	**C** * **16∶01**	**B** * **45∶01**	1	0.0021	0.3156	0.95		**C** * **08∶01**	**B** * **51∶01**		1		0.0021		−0.2468		−0.32	
	**C** * **16∶01**	**B** * **51∶01**	1	0.0021	0.0245	0.28		**C** * **16∶04**	**B** * **52∶01**		1		0.0021		0.4890		0.98	
	**C** * **07∶01**	**B** * **57∶03**	1	0.0021	1.0000	1.00		**C** * **06∶02**	**B** * **52∶01**		1		0.0021		0.0422		0.41	
	**C** * **07∶01**	**B** * **58∶01**	1	0.0021	0.2954	0.89		**C** * **04∶01**	**B** * **55∶01**		1		0.0021		0.1795		0.49	
								**C** * **0801**	**B** * **4027**		1		0.0021		0.2127		0.85	
**Total**			**26**	**0.0551**				**C** * **12∶03**	**B** * **57∶01**		1		0.0021		0.1201		0.85	
								**C** * **04∶01**	**B** * **58∶01**		1		0.0021		0.1795		0.49	
							**Total**			**110**		**0.2326**						

Blocks of each ancestry (Amerindian, Caucasian, Caucasian shared with other populations, African, and Asian) were defined as those found in original populations with H.F. >1,0%, and not found in other native human groups in frequencies higher than 1,0%. We consider *t* value must be ≥2.0 to denote statistically significant association and thus validate Δ′ (shaded values).

* Similar to B*35∶01 with a mutation at codon 207 ggc>tgc (Gly>Cys).

§ Similar to C*03∶04 with a mutation at codon 189 gtg>atg (Val>Met).

Eighteen *HLA-C/−B* blocks (13.2%) were of Caucasian MPA, the most frequent being: C*07∶02/B*07∶02 (HF = 0.0320), C*16∶01/B*44∶03 (HF = 0.0170), C*12∶03/B*38∶01 (HF = 0.0128), and C*05∶01/B*18∶01 (HF = 0.0106). The most common predominantly Caucasian *-C*/−*B* blocks shared with other ethnic groups were C*04∶01/B*35∶01 (HF = 0.0320), C*08∶02/B*14∶02 (HF = 0.0235), C*15∶02/B*51∶01 (HF = 0.0192), and C*06∶02/B*13∶02 (HF = 0.0106).

We also found 12 blocks (5.5%) from African MPA, being C*07∶01/B*49∶01 (HF = 0.0128), C*04∶01/B*53∶01 (HF = 0.0128) and C*06∶02/B*58∶02 (HF = 0.0128) were the most representative. Also 11 blocks (3.3%) of Asian MPA were found in our sample, all of them were uncommon, with frequencies below 1.0%. *HLA-C/B* blocks that were not previously reported numbered 57 (19.4%) –including two haplotypes harboring *HLA-B* and *HLA-C* new alleles-, even though the vast majority of them did not reach frequencies above 1.0%. C*01∶02/B*15∶15 (HF = 0.0277), C*01∶02/B*35∶43 (HF = 0.0192), C*15∶09/B*51∶01 (HF = 0.0192), C*03∶03/B*52∶01 (HF = 0.0129) and C*04∶01/B*35∶14 (HF = 0.0128) were the main non-previously described *HLA-C/−B* associations.

The frequencies of *HLA-DRB1/−DQB1* blocks are sumarized in **Table 3**. Eight *HLA-DRB1/−DQB1* blocks (n = 240 of 468, 51.2%) were from Amerindian MPA. The most frequent blocks were: DRB1*08∶02/DQB1*04∶02 (HF = 0.1902), DRB1*04∶07/DQB1*03∶02 (HF = 0.1153), DRB1*14∶06/DQB1*03∶01 (HF = 0.0983), DRB1*16∶02/DQB1*03∶01 (HF = 0.0641), and DRB1*14∶02/DQB1*03∶01 (HF = 0.0235). In addition, 10 Caucasian MPA and 19 predominantly Caucasian *HLA*-*DRB1/−DQB1* blocks were frequent in this sample. For example: DRB1*03∶01/DQB1*02∶01 (HF = 0.0320); DRB1*15∶01/DQB1*06∶02 (HF = 0.0320) and DRB1*04∶02/DQB1*03∶02 (HF = 0.0214) were the most frequent Caucasian MPA haplotypes in our group, while DRB1*04∶04/DQB1*03∶02 (HF = 0.0620); DRB1*07∶01/DQB1*02∶02 (HF = 0.0598); DRB1*01∶02/DQB1*05∶01 (HF = 0.0235) and DRB1*04∶03/DQB1*03∶02 (HF = 0.0214) were the most common blocks that are usually found in European populations. All these haplotypes exhibited significant LD with Δ′ values higher than 0.85. We found 8 African and seven Asian MPA blocks with frequencies lower than 1.0%. Six *-DRB1*/−*DQB1* haplotypes not found in autochthonous populations [14], including one haplotype bearing DRB1*16∶01 in association with a new DQB1*05 allele, are reported in our group. All the above mentioned *HLA-C/−B* (**Table 2**) and *HLA-DRB1/DQB1* (**Table 3**) blocks were in LD represented by significant Δ′ values and were demonstrated to be statistically relevant as they have *t* values ≥2.0 and *p* values <0.0005.

**Table 3:** Frequencies of *HLA-DRB1-DQB1* blocks in 234 Mexican admixed individuals (468 haplotypes).

	DRB1-DQB1 block		n	H.F.	Δ′	t		DRB1-DQB1 block		n	H.F.	Δ′	t
**Amerindian**	DRB1*08∶02	DQB1*04∶02	89	0.1902	0.9723	12.33	**African**	DRB1*10∶01	DQB1*05∶01	5	0.0107	0.8211	2.24
	DRB1*04∶07	DQB1*03∶02	54	0.1153	0.9518	8.51		DRB1*13∶01	DQB1*03∶03	4	0.0086	0.3942	1.96
	DRB1*14∶06	DQB1*03∶01	46	0.0983	0.9717	7.88		DRB1*08∶04	DQB1*03∶01	2	0.0043	1.0000	1.55
	DRB1*16∶02	DQB1*03∶01	30	0.0641	1.0000	6.25		DRB1*03∶02	DQB1*04∶02	1	0.0022	1.0000	1.05
	DRB1*14∶02	DQB1*03∶01	11	0.0235	1.0000	3.68		DRB1*12∶01	DQB1*05∶01	1	0.0022	0.4632	0.93
	DRB1*04∶11	DQB1*03∶02	8	0.0171	0.8526	2.93		DRB1*13∶01	DQB1*05∶01	1	0.0022	0.0159	0.18
	DRB1*04∶10	DQB1*04∶02	1	0.0022	0.3706	0.71		DRB1*13∶04	DQB1*03∶01	1	0.0022	1.0000	1.10
	DRB1*04∶11	DQB1*04∶02	1	0.0022	−0.4595	−0.70		DRB1*15∶03	DQB1*06∶02	1	0.0022	1.0000	1.00
**Total**			**240**	**0.5129**			**Total**			**16**	**0.0346**		
**Caucasian**	DRB1*03∶01	DQB1*02∶01	15	0.0320	1.0000	4.02	**Asian**	DRB1*11∶02	DQB1*03∶01	3	0.0064	0.6674	1.60
	DRB1*15∶01	DQB1*06∶02	15	0.0320	0.8779	4.01		DRB1*04∶04	DQB1*04∶02	2	0.0043	−0.6862	−2.03
	DRB1*04∶02	DQB1*03∶02	10	0.0214	1.0000	3.50		DRB1*09∶01	DQB1*03∶03	1	0.0022	1.0000	1.00
	DRB1*11∶04	DQB1*03∶01	8	0.0171	1.0000	3.13		DRB1*12∶01	DQB1*03∶01	1	0.0022	0.3348	0.63
	DRB1*13∶01	DQB1*06∶03	6	0.0128	1.0000	2.49		DRB1*13∶01	DQB1*06∶02	1	0.0022	0.0487	0.59
	DRB1*07∶01	DQB1*03∶03	5	0.0107	0.4620	2.10		DRB1*13∶02	DQB1*05∶01	1	0.0022	0.0338	0.33
	DRB1*04∶01	DQB1*03∶02	3	0.0064	1.0000	1.90		DRB1*15∶01	DQB1*05∶01	1	0.0022	−0.1415	−0.16
	DRB1*11∶01	DQB1*03∶01	2	0.0043	0.1130	0.40							
	DRB1*04∶07	DQB1*03∶01	1	0.0022	−0.9268	−3.80	**Total**			**10**	**0.0217**		
	DRB1*08∶03	DQB1*03∶01	1	0.0022	1.0000	−0.60							
**Total**			**66**	**0.1411**									
**Caucasian shared with other populations**	DRB1*04∶04	DQB1*03∶02	29	0.0620	0.9144	5.92		DRB1*04∶07	DQB1*06∶04	1	0.0022	−0.1509	−0.17
	DRB1*07∶01	DQB1*02∶02	28	0.0598	1.0000	5.66		DRB1*10∶01	DQB1*05∶02	1	0.0022	0.3247	0.98
	DRB1*01∶02	DQB1*05∶01	11	0.0235	1.0000	3.41		DRB1*11∶02	DQB1*03∶19	1	0.0022	0.2419	0.98
	DRB1*04∶03	DQB1*03∶02	10	0.0214	1.0000	3.50		DRB1*14∶06	DQB1*04∶02	1	0.0022	−0.8965	−3.37
	DRB1*01∶01	DQB1*05∶01	9	0.0192	1.0000	3.07		DRB1*16∶01	DQB1*05:New^†^	1	0.0022	1.0000	1.00
	DRB1*13∶02	DQB1*06∶04	9	0.0192	0.8978	3.07		**Total**		**7**	**0.0153**		
	DRB1*14∶01	DQB1*05∶03	8	0.0171	1.0000	2.89							
	DRB1*15∶02	DQB1*06∶01	5	0.0107	1.0000	2.27							
	DRB1*11∶01	DQB1*03∶19	4	0.0086	0.7974	2.02							
	DRB1*01∶03	DQB1*05∶01	3	0.0064	1.0000	1.75							
	DRB1*12∶02	DQB1*03∶01	3	0.0064	1.0000	1.90							
	DRB1*13∶03	DQB1*03∶01	3	0.0064	1.0000	1.90							
	DRB1*04∶05	DQB1*03∶02	1	0.0022	1.0000	1.09							
	DRB1*04∶08	DQB1*03∶01	1	0.0022	1.0000	1.10							
	DRB1*04∶10	DQB1*03∶02	1	0.0022	0.3366	0.63							
	DRB1*08∶01	DQB1*04∶02	1	0.0022	1.0000	1.05							
	DRB1*13∶05	DQB1*03∶01	1	0.0022	1.0000	1.10							
	DRB1*15∶01	DQB1*05∶02	1	0.0022	0.3081	0.93							
	DRB1*16∶01	DQB1*05∶02	1	0.0022	0.4968	1.00							
**Total**			**129**	**0.2761**									

Blocks of each ancestry (Amerindian, Caucasian, Caucasian shared with other populations, African and Asian) were defined as those found in original populations with H.F. >1,0%, and not found in other native human groups in frequencies higher than 1,0%. We consider *t* value must be ≥2.0 to denote statistically significant association and thus validate Δ′ (shaded values).^†^ Similar to DQB1*05∶02 with a silent mutation at codon 133 cgg>cga.

### Conserved Extended HLA Haplotypes

We listed known CEHs in **Table 4**. A total of 23 Amerindian, 10 Caucasian, 8 Caucasian-shared with other populations, 1 African, 1 Asian, and 37 not previously reported (unknown) *HLA-C*/−*B*/−*DRB1*/−*DQB1* haplotypes were found in our admixed Mexican sample. Amerindian CEHs with frequencies higher than 1.0% were C*07∶02/B*39∶05/DRB1*04∶07/DQB1*03∶02 (HF = 0.0406); C*07∶02/B*39∶06/DRB1*14∶06/DQB1*03∶01 (HF = 0.0342); C*04∶01/B*35∶17/DRB1*08∶02/DQB1*04∶02 (HF = 0.0299); C*01∶02/B*15∶15/DRB1*08∶02/DQB1*04∶02 (HF = 0.0171); C*08∶01/B*48∶01/DRB1*08∶02/DQB1*04∶02 (HF = 0.0171), and C*04∶01/B*35∶12/DRB1*08∶02/DQB1*04∶02 (HF = 0.015). Caucasian MPA blocks with frequencies above 1.0% include: C*07∶02/B*07∶02/DRB1*15∶01/DQB1*06∶02 (HF = 0.0150), C*16∶01/B*44∶03/DRB1*07∶01/DQB1*02∶02 (HF = 0.0128); and C*08∶02/B*14∶02/DRB1*01∶02/DQB1*05∶01 (HF = 0.0107). Neither African, Asian, Caucasian-shared with other populations, nor not previously reported haplotypes were found in frequencies above 1.0%, except for haplotype C*07∶02/B*39∶05/DRB1*08∶02/DQB1*04∶02 (HF = 0.0107), although it appears not to be in LD. *t* value for this haplotype does not reach statistical significance. For the rest of the CEHs detected, *t* values were ≥2.0.

**Table 4:** HLA Conserved Extended Haplotypes in 234 Mexican admixed individuals (468 haplotypes).

	C-B-DRB1-DQB1 haplotype				n	H.F.	Δ′	t		C-B-DRB1-DQB1 haplotype				n	H.F.	Δ′	t
**Amerindian**	C*07∶02	B*39∶05	DRB1*04∶07	DQB1*03∶02	19	0.0406	0.5025	4.15	**African**	C*06∶02	B*58∶02	DRB1*13∶01	DQB1*03∶03	4	0.0086	1.0000	2.02
	C*07∶02	B*39∶06	DRB1*14∶06	DQB1*03∶01	16	0.0342	0.5482	3.89	**Total**					**4**	**0.0086**		
	C*04∶01	B*35∶17	DRB1*08∶02	DQB1*04∶02	14	0.0299	0.7256	3.64	**Asian**	C*08∶01	B*15∶02	DRB1*12∶02	DQB1*03∶01	2	0.0043	1.0000	1.42
	C*01∶02	B*15∶15	DRB1*08∶02	DQB1*04∶02	8	0.0171	0.5251	2.45	**Total**					**2**	**0.0043**		
	C*08∶01	B*48∶01	DRB1*08∶02	DQB1*04∶02	8	0.0171	1.0000	2.23	**Unknown**	C*07∶02	B*39∶05	DRB1*08∶02	DQB1*04∶02	5	0.0107	−0.2267	−0.14
	C*04∶01	B*35∶12	DRB1*08∶02	DQB1*04∶02	7	0.0150	0.3054	1.78		C*04∶01	B*53∶01	DRB1*13∶02	DQB1*06∶04	4	0.0086	0.6601	2.01
	C*01∶02	B*15∶30	DRB1*08∶02	DQB1*04∶02	4	0.0086	0.3826	1.49		C*04∶01	B*35∶14	DRB1*16∶02	DQB1*03∶01	4	0.0086	0.6438	1.95
	C*03∶05	B*40∶02	DRB1*04∶07	DQB1*03∶02	4	0.0086	0.3234	1.61		C*01∶02	B*35∶43	DRB1*04∶03	DQB1*03∶02	4	0.0086	0.4323	1.98
	C*15∶02	B*51∶01	DRB1*08∶02	DQB1*04∶02	4	0.0086	0.3140	1.36		C*03∶04	B*40∶02	DRB1*16∶02	DQB1*03∶01	4	0.0086	0.3200	1.78
	C*03∶05	B*35∶01	DRB1*04∶07	DQB1*03∶02	3	0.0064	0.5489	1.59		C*07∶02	B*39∶06	DRB1*04∶07	DQB1*03∶02	4	0.0086	0.0394	−0.66
	C*04∶01	B*35∶17	DRB1*16∶02	DQB1*03∶01	3	0.0064	0.1096	1.14		C*01∶02	B*15∶01	DRB1*08∶02	DQB1*04∶02	3	0.0064	0.2944	1.13
	C*07∶02	B*39∶05	DRB1*16∶02	DQB1*03∶01	3	0.0064	0.0295	0.49		C*04∶01	B*35∶01	DRB1*08∶02	DQB1*04∶02	3	0.0064	0.0121	0.09
	C*03∶05	B*40∶02	DRB1*04∶04	DQB1*03∶02	3	0.0064	0.2538	1.48		C*07∶02	B*39∶05	DRB1*14∶02	DQB1*03∶01	3	0.0064	0.2158	1.37
	C*03∶03	B*52∶01	DRB1*14∶06	DQB1*03∶01	3	0.0064	0.4455	1.55		C*08∶01	B*48∶01	DRB1*04∶04	DQB1*03∶02	3	0.0064	0.1472	1.28
	C*01∶02	B*15∶01	DRB1*16∶02	DQB1*03∶01	2	0.0043	0.2368	1.17		C*15∶09	B*51∶01	DRB1*04∶07	DQB1*03∶02	3	0.0064	0.2482	1.27
	C*01∶02	B*15∶30	DRB1*14∶06	DQB1*03∶01	2	0.0043	0.1682	0.93		C*07∶02	B*07∶02	DRB1*11∶04	DQB1*03∶01	2	0.0043	0.2235	1.28
	C*04∶01	B*35∶24	DRB1*16∶02	DQB1*03∶01	2	0.0043	0.4658	1.32		C*01∶02	B*15∶01	DRB1*04∶04	DQB1*03∶02	2	0.0043	0.2385	1.18
	C*01∶02	B*35∶43	DRB1*08∶02	DQB1*04∶02	2	0.0043	0.0396	0.21		C*04∶07	B*15∶31	DRB1*14∶01	DQB1*05∶03	2	0.0043	1.0000	1.42
	C*07∶02	B*39∶01	DRB1*08∶02	DQB1*04∶02	2	0.0043	0.3826	1.05		C*05∶01	B*18∶01	DRB1*11∶01	DQB1*03∶19	2	0.0043	0.4946	1.41
	C*03∶04	B*39∶02	DRB1*04∶11	DQB1*03∶02	2	0.0043	0.3896	1.39		C*04∶01	B*35∶01	DRB1*16∶02	DQB1*03∶01	2	0.0043	0.0740	0.77
	C*07∶02	B*39∶02	DRB1*08∶02	DQB1*04∶02	2	0.0043	0.3826	1.05		C*06∶02	B*35∶02	DRB1*11∶04	DQB1*03∶01	2	0.0043	1.0000	1.42
	C*03∶04	B*40∶02	DRB1*08∶02	DQB1*04∶02	2	0.0043	−0.0439	−0.06		C*04∶01	B*35∶12	DRB1*04∶04	DQB1*03∶02	2	0.0043	0.0672	0.75
	C*08∶03	B*48∶01	DRB1*08∶02	DQB1*04∶02	2	0.0043	0.5884	1.25		C*04∶01	B*35∶12	DRB1*04∶07	DQB1*03∶02	2	0.0043	0.0133	0.14
**Total**					**117**	**0.2504**				C*04∶01	B*35∶12	DRB1*14∶06	DQB1*03∶01	2	0.0043	0.0296	0.31
										C*01∶02	B*35∶43	DRB1*04∶04	DQB1*03∶02	2	0.0043	0.1708	1.09
**Caucasian**	C*07∶02	B*07∶02	DRB1*15∶01	DQB1*06∶02	7	0.0150	0.4478	2.62		C*06∶02	B*37∶01	DRB1*01∶03	DQB1*05∶01	2	0.0043	0.6645	1.42
	C*16∶01	B*44∶03	DRB1*07∶01	DQB1*02∶02	6	0.0128	0.7341	2.44		C*03∶04	B*39∶02	DRB1*04∶04	DQB1*03∶02	2	0.0043	0.3604	1.28
	C*08∶02	B*14∶02	DRB1*01∶02	DQB1*05∶01	5	0.0107	0.4414	2.22		C*07∶02	B*39∶02	DRB1*16∶02	DQB1*03∶01	2	0.0043	0.4658	1.32
	C*06∶02	B*13∶02	DRB1*07∶01	DQB1*02∶02	4	0.0086	0.7873	1.99		C*07∶02	B*39∶05	DRB1*14∶06	DQB1*03∶01	2	0.0043	−0.4015	−0.85
	C*05∶01	B*18∶01	DRB1*03∶01	DQB1*02∶01	3	0.0064	0.5868	1.71		C*07∶02	B*39∶06	DRB1*04∶04	DQB1*03∶02	2	0.0043	0.0129	0.24
	C*07∶01	B*57∶01	DRB1*07∶01	DQB1*03∶03	3	0.0064	1.0000	1.74		C*07∶02	B*39∶08	DRB1*04∶07	DQB1*03∶02	2	0.0043	0.6241	1.33
	C*07∶01	B*08∶01	DRB1*03∶01	DQB1*02∶01	2	0.0043	0.6556	1.40		C*12∶03	B*39∶10	DRB1*07∶01	DQB1*02∶02	2	0.0043	1.0000	1.42
	C*12∶03	B*38∶01	DRB1*04∶02	DQB1*03∶02	2	0.0043	0.3188	1.37		C*03∶04	B*40∶02	DRB1*04∶03	DQB1*03∶02	2	0.0043	0.1807	1.30
	C*12∶03	B*38∶01	DRB1*07∶01	DQB1*02∶02	2	0.0043	0.2909	1.24		C*03∶04	B*40∶02	DRB1*14∶06	DQB1*03∶01	2	0.0043	0.0926	0.69
	C*05∶01	B*44∶02	DRB1*04∶02	DQB1*03∶02	2	0.0043	0.4891	1.40		C*03∶05	B*40∶02	DRB1*14∶02	DQB1*03∶01	2	0.0043	0.1807	1.30
**Total**					**36**	**0.0771**				C*07∶01	B*41∶01	DRB1*08∶04	DQB1*03∶01	2	0.0043	1.0000	1.42
										C*06∶02	B*45∶01	DRB1*07∶01	DQB1*02∶02	2	0.0043	1.0000	1.42
**Caucasian shared with other populations**	C*08∶02	B*14∶01	DRB1*07∶01	QDB1*02∶02	3	0.0064	0.7341	1.71		C*07∶02	B*51∶01	DRB1*15∶01	DQB1*06∶02	2	0.0043	1.0000	1.42
	C*07∶01	B*49∶01	DRB1*13∶02	DQB1*06∶04	3	0.0064	0.4902	1.72		C*15∶09	B*51∶01	DRB1*04∶11	DQB1*03∶02	2	0.0043	0.2353	1.35
	C*04∶01	B*35∶01	DRB1*04∶04	DQB1*03∶02	3	0.0064	0.1472	1.28		C*15∶09	B*51∶01	DRB1*08∶02	DQB1*04∶02	2	0.0043	0.0396	0.21
	C*08∶02	B*14∶02	DRB1*03∶01	DQB1*02∶01	2	0.0043	0.1547	1.22		C*03∶03	B*52∶01	DRB1*08∶02	DQB1*04∶02	2	0.0043	0.1768	0.69
	C*04∶01	B*35∶01	DRB1*11∶04	DQB1*03∶01	2	0.0043	0.2252	1.29	**Total**					**92**	**0.1975**		
	C*04∶01	B*35∶01	DRB1*14∶01	DQB1*05∶03	2	0.0043	0.2252	1.29									
	C*07∶02	B*07∶02	DRB1*15∶02	DQB1*06∶01	2	0.0043	0.3788	1.35									
	C*06∶02	B*50∶01	DRB1*03∶01	DQB1*02∶01	2	0.0043	0.4834	1.38									
**Total**					**19**	**0.0407**											

### Extension of Conserved Extended HLA Haplotypes to HLA-A

In **Table 5** we show the preferential association of the common *HLA-C/−B/−DRB1/−DQB1* CEH with *HLA-A* alleles in Mexican admixed population. It is remarkable that CEHs were common in this sample, with five haplotypes found with HF ≥1.0%: A*24∶02/C*07∶02/B*39∶06/DRB1*14∶06/DQB1*03∶01 (HF = 0.0256), A*02∶01/C*04∶01/B*35∶17/DRB1*08∶02/DQB1*04∶02 (HF = 0.0150), A*68∶03/C*07∶02/B*39∶05/DRB1*04∶07/DQB1*03∶02 (HF = 0.0107), A*02∶06/C*07∶02/B*39∶05/DRB1*04∶07/DQB1*03∶02 (HF = 0.0107), and A*02∶01/C*07∶02/B*39∶05/DRB1*04∶07/DQB1*03∶02 (HF = 0.0128), the first four of them being identified within samples of Native American people from all over the Americas, and the last one not found yet in other populations. Importantly, six Caucasian and one African CEHs were found. A set of 38 haplotypes was classified as not previously reported (unknown), some of them resulted from recombination between Caucasian and Amerindian blocks. Interestingly, one CEH which is frequent in Askenazi Jewish population was also observed in our sample (A*26∶01/C*12∶03/B*38∶01/DRB1*04∶02/DQB1*03∶02).

**Table 5:** Extension of HLA Conserved Extended Haplotypes to *HLA-A* in 234 Mexican Admixed individuals.

	A-C-B-DRB1-DQB1 haplotype					n	H.F.	Δ′	t
**Amerindian**	A*24∶02	C*07∶02	B*39∶06	DRB1*14∶06	DQB1*03∶01	12	0.0256	0.6992	2.76
	A*02∶01	C*04∶01	B*35∶17	DRB1*08∶02	DQB1*04∶02	7	0.0150	0.3518	1.46
	A*68∶03	C*07∶02	B*39∶05	DRB1*04∶07	DQB1*03∶02	5	0.0107	0.2834	2.02
	A*02∶06	C*07∶02	B*39∶05	DRB1*04∶07	DQB1*03∶02	5	0.0107	0.1848	1.46
	A*02∶01	C*04∶01	B*35∶12	DRB1*08∶02	DQB1*04∶02	4	0.0086	0.4444	1.21
	A*02∶01	C*01∶02	B*15∶15	DRB1*08∶02	DQB1*04∶02	3	0.0064	0.1898	0.68
	A*68∶01	C*01∶02	B*15∶15	DRB1*08∶02	DQB1*04∶02	3	0.0064	0.3214	1.38
	A*02∶01	C*08∶01	B*48∶01	DRB1*08∶02	DQB1*04∶02	3	0.0064	0.1898	0.68
	A*02∶06	C*08∶01	B*48∶01	DRB1*08∶02	DQB1*04∶02	3	0.0064	0.3085	1.31
	A*24∶02	C*04∶01	B*35∶12	DRB1*08∶02	DQB1*04∶02	2	0.0043	0.1407	0.58
	A*68∶01	C*04∶01	B*35∶17	DRB1*08∶02	DQB1*04∶02	2	0.0043	0.0693	0.64
	A*02∶06	C*03∶04	B*40∶02	DRB1*08∶02	DQB1*04∶02	2	0.0043	1.0000	1.28
	A*02∶06	C*04∶01	B*35∶17	DRB1*08∶02	DQB1*04∶02	2	0.0043	0.5170	0.47
	A*02∶01	C*03∶04	B*39∶02	DRB1*04∶04	DQB1*03∶02	2	0.0043	1.0000	1.10
	A*02∶01	C*07∶02	B*39∶05	DRB1*16∶02	DQB1*03∶01	2	0.0043	0.5679	0.93
	A*31∶01	C*04∶01	B*35∶17	DRB1*08∶02	DQB1*04∶02	2	0.0043	0.0693	0.64
**Total**						**59**	**0.1263**		
**Caucasian**	A*02∶01	C*07∶02	B*07∶02	DRB1*15∶01	DQB1*06∶02	4	0.0086	0.4444	1.21
	A*30∶02	C*05∶01	B*18∶01	DRB1*03∶01	DQB1*02∶01	3	0.0064	1.0000	1.72
	A*29∶02	C*16∶01	B*44∶03	DRB1*07∶01	DQB1*02∶02	2	0.0043	0.3158	1.32
	A*02∶01	C*16∶01	B*44∶03	DRB1*07∶01	DQB1*02∶02	2	0.0043	0.1357	0.45
	A*01∶01	C*07∶01	B*57∶01	DRB1*07∶01	DQB1*03∶03	2	0.0043	0.6541	1.35
	A*33∶01	C*08∶02	B*14∶02	DRB1*01∶02	DQB1*05∶01	2	0.0043	0.3922	1.38
**Total**						**15**	**0.0322**		
**African**	A*66∶01	C*06∶02	B*58∶02	DRB1*13∶01	DQB1*03∶03	4	0.0086	1.0000	1.99
			**Total**			**4**	**0.0086**		
**Unknown**	A*02∶01	C*07∶02	B*39∶05	DRB1*04∶07	DQB1*03∶02	6	0.0128	0.1130	0.68
	A*24∶02	C*04∶01	B*35∶14	DRB1*16∶02	DQB1*03∶01	4	0.0086	1.0000	1.67
	A*30∶01	C*06∶02	B*13∶02	DRB1*07∶01	DQB1*02∶02	4	0.0086	1.0000	1.99
	A*68∶02	C*04∶01	B*53∶01	DRB1*13∶02	DQB1*06∶04	4	0.0086	1.0000	1.96
	A*02∶01	C*01∶02	B*15∶30	DRB1*08∶02	DQB1*04∶02	3	0.0064	0.6759	1.21
	A*23∶01	C*07∶01	B*41∶01	DRB1*08∶04	DQB1*03∶01	2	0.0043	1.0000	1.40
	A*66∶01	C*12∶03	B*39∶10	DRB1*07∶01	DQB1*02∶02	2	0.0043	1.0000	1.40
	A*26∶01	C*06∶02	B*37∶01	DRB1*01∶03	DQB1*05∶01	2	0.0043	1.0000	1.39
	**Table 5** **.**	**Cont.**							
	A*26∶01	C*06∶02	B*45∶01	DRB1*07∶01	DQB1*02∶02	2	0.0043	1.0000	1.39
	A*03∶01	C*06∶02	B*35∶02	DRB1*11∶04	DQB1*03∶01	2	0.0043	1.0000	1.38
	A*68∶01	C*07∶02	B*39∶01	DRB1*08∶02	DQB1*04∶02	2	0.0043	1.0000	1.31
	A*68∶01	C*07∶02	B*39∶02	DRB1*16∶02	DQB1*03∶01	2	0.0043	1.0000	1.31
	A*31∶01	C*03∶03	B*52∶01	DRB1*08∶02	DQB1*04∶02	2	0.0043	1.0000	1.31
	A*02∶06	C*01∶02	B*15∶30	DRB1*14∶06	DQB1*03∶01	2	0.0043	1.0000	1.28
	A*02∶06	C*04∶01	B*35∶24	DRB1*16∶02	DQB1*03∶01	2	0.0043	1.0000	1.28
	A*31∶01	C*03∶05	B*35∶01	DRB1*04∶07	DQB1*03∶02	2	0.0043	0.6381	1.25
	A*31∶01	C*03∶05	B*40∶02	DRB1*04∶04	DQB1*03∶02	2	0.0043	0.6381	1.25
	A*02∶06	C*15∶09	B*51∶01	DRB1*04∶07	DQB1*03∶02	2	0.0043	0.6312	1.22
	A*31∶01	C*03∶04	B*40∶02	DRB1*04∶07	DQB1*03∶02	2	0.0043	0.4571	1.20
	A*31∶01	C*03∶05	B*40∶02	DRB1*16∶02	DQB1*03∶01	2	0.0043	0.4571	1.20
	A*24∶02	C*04∶01	B*35∶01	DRB1*14∶01	DQB1*05∶03	2	0.0043	1.0000	1.18
	A*24∶02	C*04∶01	B*35∶12	DRB1*04∶04	DQB1*03∶02	2	0.0043	1.0000	1.18
	A*24∶02	C*01∶02	B*35∶43	DRB1*04∶04	DQB1*03∶02	2	0.0043	1.0000	1.18
	A*24∶02	C*03∶04	B*40∶02	DRB1*14∶06	DQB1*03∶01	2	0.0043	1.0000	1.18
	A*02∶06	C*03∶04	B*40∶02	DRB1*16∶02	DQB1*03∶01	2	0.0043	0.4468	1.15
	A*02∶01	C*07∶02	B*07∶02	DRB1*11∶04	DQB1*03∶01	2	0.0043	1.0000	1.10
	A*02∶01	C*01∶02	B*15∶01	DRB1*04∶04	DQB1*03∶02	2	0.0043	1.0000	1.10
	A*02∶01	C*01∶02	B*35∶43	DRB1*08∶02	DQB1*04∶02	2	0.0043	1.0000	1.10
	A*02∶01	C*03∶04	B*39∶02	DRB1*04∶11	DQB1*03∶02	2	0.0043	1.0000	1.10
	A*02∶01	C*05∶01	B*44∶02	DRB1*04∶02	DQB1*03∶02	2	0.0043	1.0000	1.10
	A*02∶01	C*08∶03	B*48∶01	DRB1*08∶02	DQB1*04∶02	2	0.0043	1.0000	1.10
	A*02∶01	C*15∶09	B*51∶01	DRB1*08∶02	DQB1*04∶02	2	0.0043	1.0000	1.10
	A*24∶02	C*01∶02	B*15∶01	DRB1*08∶02	DQB1*04∶02	2	0.0043	0.5990	1.06
	A*24∶02	C*07∶02	B*39∶06	DRB1*04∶07	DQB1*03∶02	2	0.0043	0.3985	0.94
	A*02∶01	C*03∶03	B*52∶01	DRB1*14∶06	DQB1*03∶01	2	0.0043	0.5679	0.93
	A*02∶01	C*04∶01	B*35∶01	DRB1*08∶02	DQB1*04∶02	2	0.0043	0.5679	0.93
	A*02∶01	C*07∶02	B*39∶05	DRB1*14∶02	DQB1*03∶01	2	0.0043	0.5679	0.93
	A*24∶02	C*07∶02	B*39∶05	DRB1*08∶02	DQB1*04∶02	2	0.0043	0.2782	0.82
**Total**						**87**	**0.1869**		

Blocks of each ancestry (Amerindian, Caucasian, Caucasian shared with other populations, African and Asian) were defined as those found in original populations with H.F. >1,0%, and not found in other native human groups in frequencies higher than 1,0%. We consider *t* value must be ≥2.0 to denote statistically significant association and thus validate Δ′ (shaded values).

### HLA Genetic Diversity in Mexicans

The extensive polymorphism of the HLA loci in this group of Mexicans was confirmed using polymorphism information content (PIC) values >0.5. *HLA-B* and *-DRB1* loci were the most polymorphic with PIC values of 0.9544 and 0.9123, respectively. *HLA-C* and *–A* loci were relatively less polymorphic with PIC values of 0.8845 and 0.8776, respectively, and the less polymorphic locus was *HLA-DQB1* (PIC = 0.8020). The degree of polymorphism of HLA loci was also corroborated by the power of discrimination (PD) values. A lower observed heterozygosity (OH) than expected heterozygosity (EH) was found for HLA-DRB1 locus, **Table 6**.

**Table 6:** Measures of genetic diversity at the allele level for HLA system in a Mexican admixed population.

HLA Allele	O.H.	E.H.	*p* value	PIC	PD
***HLA-A***	0.8718	0.8919	0.5303	0.8776	0.7382
***HLA-B***	0.9487	0.9668	0.2942	0.9544	0.8531
***HLA-C***	0.9009	0.8947	0.2001	0.8845	0.7972
***HLA-DRB1***	0.9013	0.9193	0.0061	0.9123	0.7981
***HLA-DQB1***	0.8205	0.8256	0.2682	0.8020	0.6376

O.H.: Observed heterozygosity. E.H.: Expected Heterozygosity. *p* values <0.05 are considered statistically significative and thus reflect differences between O.H. and E.H. PIC: Polymorphism Information Contents. PD: Power of Discrimination.

### Mexican Admixed Individuals have a Significant Proportion of Amerindian and Caucasian Genetic Components

The admixture estimations using *HLA-B* revealed an Amerindian contribution of 59.97%; Caucasian contribution of 25.71%; African contribution of 14.13%; and Asian contribution of 0.18%. These results were similar to the estimations obtained using STRs: Amerindian contribution: 60.5%; Caucasian: 25.9% and African: 13.6%.

**Figure 1:**
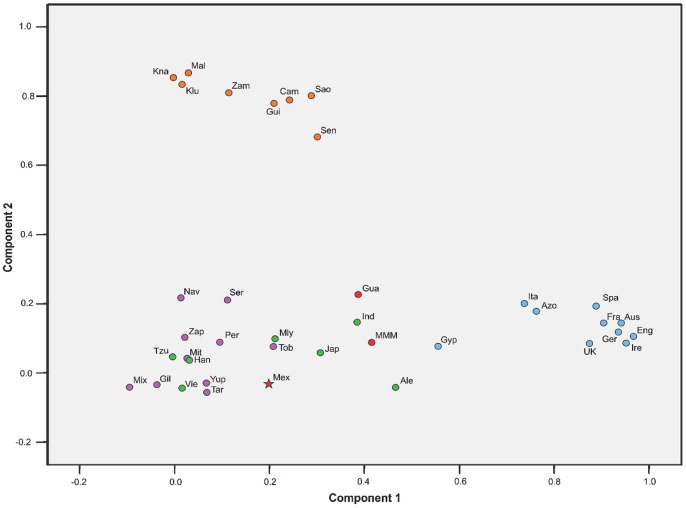
Principal component analysis (PCA) plot reveals a close genetic relationship of Mexican admixed individuals from Mexico City to Native American groups. Orange dots refer to African populations. Blue dots represent European samples. Green dots correspond to Asian human groups. Native American populations are represented by purple dots. Red figures are admixed populations from Mexico, with a star locating our Mexico City admixed sample. Proper references of each population group included in the analysis are given in the Materials and Methods section. Ire: Ireland, Eng: England, Ger: Germany, Aus: Austria; Spa: Spain, Ita: Italy, UK: United Kingdom, Fra: France, Gyp: Gypsy, Azo: Azores, Sao: São Tomé Island, Cam: Cameroon, Mal: Mali, Zam: Zambia, KLu: Luo from Kenia, KNa: Nandi from Kenia, Sen: Senegal, Gui: Guinea Bissau, Ale: Aleut from Bering Island, Jap: Japan, Tzu: Taiwan, Han: south China, Ind: north India, Mly: Malasya, Vie: Vietnam, Tar: Tarahumara, Gil: Native Americans from Gila River, Yup: Yu’pik from Alaska, Mit: Mixtec from Oaxaca, Zap: Zapotec from Oaxaca, Mix: Mixe from Oaxaca, Ser: Seri from Sonora, Nav: Navajo from New Mexico, Uro: Uro from Titikaka Lake, Tob: Toba from Rosario, MMM: “Mexican Mestizo” sample, Gua: Guadalajara City, Mex: this study.

In addition, the results using the ABF revealed a frequency of Amerindian *HLA-C*/−*B* blocks of 41.3%, followed by Caucasian 25.8%, African 5.5% and Asian 3.3% blocks. The ABF of MHC class II blocks were as follows: Amerindian 51.2%, Caucasian 41.7%, African 3.4% and Asian 2.1%. Further evidence of the distribution of immunogenetic diversity can be observed in the principal component analysis (PCA) plot (**Figure 1**), in which our Mexican admixed sample (Mex) clusters together with Native American and Asian populations (which can not be clearly differentiated from each other when HLA-B frequencies are taken as the variable of the factor analysis), and not with the African or European clusters.

## Discussion

Here, we analyzed MHC class I (*HLA-C/B)* and class II (*HLA-DRB1/DQB1)* blocks diversity, ancestry, and the frequency of CEHs from *HLA-C/B/DRB1/DQB1* and their extension to *HLA-A* in a total number of 468 haplotypes of individuals from Mexico City. We found that 41.0% of the *HLA*-*C/−B* blocks in our group were from Amerindian origin. In addition, some of these *HLA*-*C*/−*B* blocks also have been described in Asian populations (e.g: C*08∶01/B*48∶01) including Ivatan from Philippines [15] and several ethnic groups from Taiwan [14]. These findings may indicate that those haplotypes could be frequent in an ancestral group from which both Amerindians and South-East Asians originated from. Amerindian *HLA*-*C*/−*B* blocks observed in the present study, have been also reported, with high frequencies, among Amerindian groups including Zapotecs, Mixe, and Mixtec from Oaxaca State in the southeast of Mexico [16]; Tarahumara from Chihuahua State in the north of Mexico [17]; Native Americans from US [18]; and Yucpa from Venezuela [19]. Genetic admixture estimations were similar to those previously reported data from Mexico City [9]. We detected 13.2% of haplotypes of Caucasian MPA and 11.1% were predominantly Caucasian but shared with other populations including the haplotypes C*07∶02/B*07∶02, C*16∶01/B*44∶03, C*12∶03/B*38∶01 and C*05∶01/B*18∶01 [13], [18], [20], [21], [22].

In the PCA, our Mexican admixed sample (Mex) clearly separated from the European and African clusters and located within a loose cluster including populations from Asia and Native human groups from America. Notably, the “Mestizo” sample from Mexico (MMM) and the sample from Guadalajara (Gua) showed to be more proximate to the European cluster; Guadalajara population samples have shown a high degree of European genetic component in other works [23], [24]. Differences in admixed populations show the importance of not taking “Mestizo” as a global grouping category for individuals or populations with shared ancestry derived from demographic history of the colonial period. Also, lack of available data with high resolution HLA typing is evident in Native American groups.

Regarding MHC class II blocks, 51.2% of them were from Amerindian MPA, the most common being DRB1*08∶02/DQB1*04∶02 and DRB1*04∶07/DQB1*03∶02, whereas 40% of the *-DRB1*/−*DQB1* blocks were from Caucasian MPA. These haplotypes are common in Mexican Amerindians, as well as in Xavante from Central Brazil, Toba from Argentina, Athabaskan from Canada, and Mayans from Guatemala [25]–[28]. Interestingly, HLA class II blocks show a restricted diversity as it was pointed out by the fact that eleven CEH (HF >0.5%; *t* ≥2.0) are associated with only three HLA class II blocks: DRB1*04∶07/DQB1*03∶02, DRB1*14∶06/DQB1*03∶01, and mainly DRB1*08∶02/DQB1*04∶02. This trend is also shown in CEH extended to the *HLA-A* locus. Less than 5% of class II blocks from African or Asian probable ancestry were detected.

Genetic diversity parameters confirm the high degree of polymorphism of the HLA genes in the studied sample. *HLA-B* and *HLA-DRB1* were the most polymorphic loci according to PIC and PD values, followed by *HLA-C* locus. However, lower OH than EH was found for *HLA-DRB1* locus. This may indicate that selective forces are acting on the *HLA-DRB1* locus in Mexicans, as well as in the Mexican Amerindian populations, resulting in low class II diversity. Also, low class II diversity may have been produced by the limited *-DRB1* allelic diversity that the first human settlers carried with them into the Americas [29]–[31] and their incorporation into the admixed Mexican genetic pool. Deviation from neutral expectations tends to ocurr by an excess of heterozygotes; however, homozygous excess has also been observed [31]. Migration patterns into Mexico City in the last 60 years also have to be taken into account to adecuately address an explanation for the low number of heterozygous individuals, as they represent an important source of incorporation of alleles and haplotypes –mainly from indigenous populations-, hence modifying the allelic diversity.

In our study the admixture estimations using STRs confirm the greater contribuition of Amerindian and Caucasian and a small contribution of African and Asian genes. The results obtained using the ABF of *HLA-C*/−*B* blocks also demonstrated a greater contribution of Amerindian (41.3%), followed by Caucasian (24.6%), African (6.7%), and Asian (3.0%) genes in the admixed Mexicans. Also, the estimations using the ABF of MHC class II blocks revealed that 51.2% of them were from Amerindian and 40.4% from Caucasian MPA. These findings suggest that ABF method is applicable to analyze the genetic diversity and ancestral structure of admixed populations. In this perspective, the genetic admixture of Mexicans could have resulted from the Spaniards, which arrived to Mexico early in the 16^th^ century. Caucasian component consisted in conquerors and colonizers from Andalucía, Leon, Extremadura, and the Castillas, as well as Portugal and Genoa. Spaniards settled extensively all over the Viceroyalty of the New Spain and a massive migration of colonizers begun on the 17^th^ century and prevailed through the next two centuries. Presence of Caucasian-MPA or Caucasian-shared blocks or haplotypes may be explained by these demographic traits. The preponderance of haplotypes commonly found in Caucasian populations may be due to the fact that more Caucasian human groups than African or Asian ones have been studied, or may simply reflect a lower genetic diversity among Caucasians. Another hypothesis is that population replacement, together with the collapse of Native American groups that took place due to infectious diseases [32] and the conquest wars, may explain the high prevalence of Caucasian genetic blocks within Mexican admixed individuals [33]. African contribution, although subtle, is present in admixed Mexicans due to slaves introduced to Mexico from Africa during the first three centuries of Spanish colonial domination. All African specific associations present in this study are found in Sub Saharan Africa [14], [18], [34,], the place where slaves were extracted from by colonial slave traders [18], [35]. For example, C*07∶01/B*49∶01, C*04∶01/B*53∶01, C*06∶02/B*58∶02, DRB1*13∶01/DQB1*03∶03, and DRB1*08∶04/DQB1*03∶01 blocks have been found in Africa, for instance in Bandiagara from Mali, Bantu from Congo, Bioko from Equatorial Guinea, Luo and Nandi from Kenya, Lusaka from Zambia, Ugandans and Kampala from Uganda, and Yaounde from Cameroon, [18], [36]–[39] and have been reported also in African American population from the US [40].

On the other hand, the presence of Asian genes in Mexican population possibly resulted from relative recent immigration of Chinese traders and slaves by transpacific travels from the oriental shores of Asia to the western coasts of Mexico, mainly disembarking in the port of Acapulco. Thus, the *Não de China* (the Manila Galleon) route, together with a foreign investment policy starting in the 19^th^ century, helped the Chinese community to become the largest non-Spaniard community in Mexico by mid-1920s [41]. The Asian contribution to the genetic pool conformation of Mexico is modest, mainly due to lack of admixture between Asian immigrants and Mexicans; however, classical Asian associations were found in our sample such as C*04∶01/B*35∶16 [42] and C*08∶01/B*15∶02 [18], [27], [43]. The admixture estimations using different indicators support a tryhybrid model of Amerindian, Caucasian and African ancestry in Mexicans. But we were able to detect also a small Asian component in Mexicans.

It is well known that MHC diversity influences the susceptibility or resistance to a wide variety of autoimmune disorders and infectious diseases caused by viruses, yeasts, bacteria and parasites. It has been suggested that pathogen-mediated selection might explain the maintenance of MHC diversity at population level [44], [45]. However, the role of MHC diversity associated to the admixture between different ethnic groups in the resistance or susceptibility to autoimmune or infectious disease remains unclear. Furthermore, recent studies have suggested that genes that confer susceptibility to autoimmune diseases might be maintained in specific ethnic groups because they primarily confer protection against infectious agents, the major factor driving selection and influencing human adaptation to local environments [2]–[6], [46]–[48]. Functional studies are necessary to define whether the genetic diversity of HLA is influenced in pathogen-enriched environments. The analyses of HLA diversity in the context of pathogen richness have shown a positive correlation between HLA class I allele diversity and pathogen richness and a negative correlation of HLA class II diversity, particularly *HLA-DQB1* loci, and pathogen richness, suggesting that HLA class I and class II genes have disctint evolutionary strategies to confer immunity against infectious agents [5]. In this context, the higher diversity of HLA class I genes may result from the high mutation rate of intracelular pathogens, particularly viruses. In contrast, the lower diversity of MHC class II genes might result from the fixation of some alleles that provide efficient immune protection against highly prevalent extracelular pathogens in specific populations (e.g. parasites). In Mexicans, we found a high frequency of some MHC class II alleles that predispose to rheumatoid arthritis (RA) (DRB1*04∶04, DRB1*14∶02, and DRB1*01∶02), to systemic lupus erythematosus (SLE) (DRB1*03∶01) [11] and to systemic sclerosis (SSc) (DRB1*11∶04) (Rodriguez-Reyna TS et al., Unpublished data). It is possible that class II MHC alleles associated with autoimmunity, together with alleles found in Native American populations may have increased their frequencies due to past selective processes or infectious and parasitic diseases developed in different environments and thus explain in part the susceptiblity to develop autoimmune diseases in Mexico or the clinical characteristics of these diseases in Mexican population.

In summary, Mexican admixed individuals from the central area of Mexico have an important component of Amerindian and Caucasian MHC class I (*HLA-C*/−*B*) and class II (*HLA-DRB1*/−*DQB1*) blocks and HLA CEHs. A relatively low frequency of African and Asian HLA blocks and CEHs were detected. In line with these results, admixture estimations using STRs and *HLA-B* revealed a greater proportion of Amerindian, followed by Caucasian and African ancestry in this population. The high frequency of certain relatively fixed haplotypes might result from many possible mechanisms, including recent population bottlenecks, recombination suppression, preferential transmission, migration and admixture, and/or genetic drift or natural selection. Our findings suggest that the study of HLA class I and class II blocks and CEHs diversity might be useful to characterize the ancestral contributions in admixed populations, as well as to perform studies of disease susceptibility and transplantation.

## Materials and Methods

### Subjects

A total of 234 unrelated Mexican admixed individuals were studied, including a group of 80 Mexican admixed participants belonging to 40 families. A total number of 468 haplotypes were analyzed in this study. Every participant came from Mexico City and had a Mexican ancestry whose parents and grandparents were born in Mexico. Age mean of studied individuals was 38.2±15.3 years. There were 120 females (51%) and 114 males (49%).

### Ethics Statement

The Institutional Review Board of the National Institute of Respiratory Diseases (INER) reviewed and approved the protocols for genetic studies. All subjects provided written informed consent for these studies, and they authorized the storage of their DNA samples at INER repositories for this and future studies. In this study we did not collected samples from minors/children, only young adults older than 17 years were included.

### HLA Typing

Genomic DNA was obtained from peripheral blood mononuclear cells (PBMC), using the QIAamp DNA mini kit (*Qiagen, Valencia, CA, USA*). High-resolution HLA class I and class II typing was performed by a sequence-based method (SBT) as previously described [49]. Briefly, we amplified exon 2 and 3 from *HLA-A*, *-B* and *-C* and exon 2 for *HLA-DRB1* and *-DQB1*. Polymerase chain reaction (PCR) contained 1.5 mM KCl, 1.5 mM MgCl_2_, 10 mM Tris–HCl (pH = 8.3), 200 mM concentrations of each dATP, dTTP, dGTP, and dCTP; 10 pM concentration of each primer, 30 ng of DNA and 0.5 U of *Taq* DNA polymerase in a final volume of 25 µl. Amplification was done on a PE9700 thermal cycler (*Applied Biosystems, Foster City, CA, USA*) using the following cycling conditions: 95°C for 30 s, 65°C for 30 s, 72°C for 1 min, preceded by 5 min at 95°C, and followed by a final elongation at 72°C for 5 min. Amplified products were sequenced independently in both directions using *BigDye Terminator* ™ chemistry in an ABI PRISM® 3730xl Genetic Analyzer (*Applied Biosystems, Foster City, CA, USA*). Data were analyzed using match tools allele assignment software (*Applied Biosystems, Foster City, CA,USA*) using the IMGT/HLA sequence database alignment tool (http://www.ebi.ac.uk/imgt/hla/align.html) [50]. Ambiguities were solved using group-specific sequencing primers (GSSPs) that have been reported and validated previously [49].

### HLA Blocks and Conserved Extended Haplotypes Assignment

HLA allelic and haplotypic frequencies were obtained by gene counting; one hundred and sixty of the 468 haplotypes were obtained by direct observation because they were obtained by HLA typing in the parents and siblings of 40 families, while the rest were acquired from HLA genotyping of 154 non-related individuals. Haplotypes were estimated by maximum likelihood methods using the computer program Arlequin ver. 3.0 [51]. This software was also used to calculate HWE, OH, and EH at a locus-by-locus level with 1×10^6^ steps in the Markov chain and 1×10^5^ dememorization steps. *p*-values ≤0.05 indicated statistical difference between OH and EH and thus a deviation from HWE. Listed *HLA-C/B*, *HLA-DRB1/DQB1* and CEHs and their extension to the *HLA-A* locus of Mexican origin were estimated by the maximum likelihood method based on the Δ′ between alleles of two loci and between the two blocks and/or the extension to the *HLA-A* region, as previously described [18]. Haplotypes or DNA blocks of African, Asian and Caucasian MPA were assigned based on previous reported frequencies [7], [13], [18]. Estimation of delta (Δ) and relative delta (Δ′) values to measure LD, nonrandom association of alleles at two or more loci, and their statistical significance, were calculated using previously described methods [18]. Absolute Δ′ values of 1 indicates complete LD; 0 corresponds to no LD. As many of this associations may return |Δ′| values of 1.000 -even though that value may be result of a random association between two infrequent alleles- we used the statistic parameter *t*, to validate all Δ′ data adjusted by sample size and number of times that each allele appeared in the sample [52]. Only *t* values ≥2.0 were considered significant.

### HLA Genetic Diversity Calculations

Genetic diversity of each HLA loci was assessed by two previously described forensic parameters: PIC and PD [53]–[55] that were computed using the PowerStat ver.1.2 spreadsheet (*Promega Corporation, Fitchburg, WI, USA*) as described elsewhere [56]. PIC measures the strength of a genetic marker for linkage studies by indicating the degree of polymorphism of a locus. PIC >0.5 is considered as highly polymorphic [54]. PD is defined as the probability of finding two random individuals with different genotypes for that locus in the studied population, and values higher than 0.8 indicate high polymorphism in the studied population context [57]. The OH and EH of all HLA loci was also calculated [53].

### Admixture Estimations in Mexicans using HLA Genes

Admixture estimates were obtained by maximum-likelihood method using the *Leadmix* software [58], with *k* = 4 parental populations (Africa, America, Asia, and Europe) and *HLA-B* as the genetic estimator. Caucasian component was estimated with a pooled sample (N = 315) consisting of data from southern Portugal [34] and an European population sample from USA [18]; African Nandi from Kenia [59] served as the African parental component (N = 239); a pooled Native American sample (N = 146) was used, which consisted of data from Mixtec of Oaxaca, SE Mexico [16] and Tarahumara from Chihuahua, north of Mexico [17]; finally, Han from southern China data (N = 281) were used to estimate the Asian contribution [60]. Principal Components Analysis (PCA) for 38 populations with HLA-B data available was performed using the IBM SPSS Statistics 19 software (*IBM Corporation, Armonk, NY, USA*) to analyse the distribution of HLA-B alleles in human groups of the proposed ancestries, **Figure 1**. PCA included population data of Ireland [20], NW of England [61], Germany [62], Austria [63]; Spain, Italy, United Kingdom [64], France [65], Gypsy from Andalucía (Spain; data collected by López-Nevot *et al.*) [14], Azores Terceira Island [66], Forro from São Tomé Island [67], Beti from Cameroon [68], Bandiagara from Mali, Lusaka from Zambia, Luo and Nandi from Kenia [69], Mandeka from Senegal [70], Guinea Bissau [71], Aleut from Bering Island (Russia) [72], center of Japan [73], a cord blood bank of Tzu Chi Foundation (Taiwan) [74], Han from southern China [60], north India [75], Kensiu from Malasya [76], Kinh from Vietnam [77], Tarahumara from northern Mexico [17], Native Americans from Gila River (USA) [78], Yu’pik from Alaska (USA) [79], Mixtec, Zapotec, and Mixe from Oaxaca (Mexico) [16], Seri from Sonora (Mexico) [80], Navajo from New Mexico (USA) [81], Uro from Titikaka Lake (Peru) [82], and Toba from Rosario (Argentina; data collected by Cintia Marcos *et al.*) [14]. Also, two admixed populations from Mexico were included: a “Mexican Mestizo” sample [83] and a sample from Guadalajara City, western Mexico [23]. As an approach to estimate the diversity and contribution of previously described [7], [13] Caucasian, Asian, and African HLA blocks in our population, we also calculated the aggregate block frequencies (ABF) [7], [13] adding the frequencies of those HLA clas I and II blocks with frequencies greater than 1% in our study population.

### Admixture Estimations in Mexicans using STRs

We genotyped fifteen autosomal STR markers (*CSF1PO, FGA, THO1, TPOX, VWA, D3S11358, D5S818, D7S820, D8S1179, D13S317, D16S539, D18S51, D21S11, D19S433*, and *D2S1338*) along with amelogenin using the Applied Biosystems AmpF*l* STR Identifiler Kit (*Applied Biosystems, Foster City, CA, USA*). PCR amplification was carried out on a Gene Amp 7500 thermocycler (*Applied Biosystems, Foster City, CA, USA*) using 1 ng of DNA according to the manufacturer’s protocol. The PCR conditions were: 95°C during 11 min followed by 28 cycles of 94°C for 1 min, 59°C for 1 min, 72°C for 1 min followed by a hold at 60°C for 60 min. PCR products were diluted 1∶15 in *Hi-Di*™ formamide and GS500-LIZ internal size standard (*Applied Biosystems, Foster City, CA, USA*) and analyzed on the ABIPrism 3100 Genetic Analyzer (*Applied Biosystems, Foster City, CA, USA*). Allele calls were made using Genotype 3.7 software by comparison with kit allelic ladders (*Applied Biosystems, Foster City, CA, USA*). We performed an admixture estimation using the STR’s data by a model-based clustering method with the *Structure* software v. 2.3.4 [84], assuming *k* = 3 populations and 1×10^4^ dememorisation steps, using Spaniards [85], Fang Africans [86], and a Native-American pool of Huastecos [87] and Tepehuas [88] from the central region of Mexico, as parental populations.
